# Impact of aldosterone-producing cell clusters on diagnostic discrepancies in primary aldosteronism

**DOI:** 10.18632/oncotarget.25418

**Published:** 2018-05-25

**Authors:** Mitsuhiro Kometani, Takashi Yoneda, Daisuke Aono, Shigehiro Karashima, Masashi Demura, Koshiro Nishimoto, Masakazu Yamagishi, Yoshiyu Takeda

**Affiliations:** ^1^ Division of Endocrinology and Hypertension, Department of Cardiovascular and Internal Medicine, Kanazawa University Graduate School of Medicine, Kanazawa, Ishikawa 920-8641, Japan; ^2^ Program Management Office for Paradigms Establishing Centers for Fostering Medical Researchers of the Future, Kanazawa University, Kanazawa, Ishikawa 920-8641, Japan; ^3^ Department of Hygiene, Kanazawa University Graduate School of Medicine, Kanazawa, Ishikawa 920-8641, Japan; ^4^ Department of Uro-Oncology, Saitama Medical University International Medical Center, Hidaka 350-1241, Japan

**Keywords:** primary aldosteronism, adrenal vein sampling, aldosterone-producing cell clusters

## Abstract

Adrenocorticotropic hormone (ACTH) stimulation is recommended in adrenal vein sampling (AVS) for primary aldosteronism (PA) to improve the AVS success rate. However, this method can confound the subtype diagnosis. Gene mutations or pathological characteristics may be related to lateralization by AVS. This study aimed to compare the rate of diagnostic discrepancy by AVS pre- versus post-ACTH stimulation and to investigate the relationship between this discrepancy and findings from immunohistochemical and genetic analyses of PA. We evaluated 195 cases of AVS performed in 2011–2017. All surgical specimens were analyzed genetically and immunohistochemically. Based on the criteria, AVS was successful in 158 patients both pre- and post-ACTH; of these patients, 75 showed diagnostic discrepancies between pre- and post-ACTH. Thus, 19 patients underwent unilateral adrenalectomy, of whom 16 had an aldosterone-producing adenoma (APA) that was positive for CYP11B2 immunostaining. Of them, 10 patients had discordant lateralization between pre- and post-ACTH. In the genetic analysis, the rate of somatic mutations was not significantly different between APA patients with versus without a diagnostic discrepancy. In the immunohistochemical analysis, CYP11B2 levels and the frequency of aldosterone-producing cell clusters (APCCs) in APAs were almost identical between patients with versus without a diagnostic discrepancy. However, both the number and summed area of APCCs in APAs were significantly smaller in patients with concordant results than in those whose diagnosis changed to bilateral PA post-ACTH stimulation. In conclusion, lateralization by AVS was affected by APCCs in the adjacent gland, but not by APA-related factors such as somatic gene mutations.

## INTRODUCTION

Primary aldosteronism (PA) is a major cause of secondary hypertension that is associated with severe cardiovascular complications [1]. Therefore, appropriate therapy is required. PA is divided into two subtypes: unilateral PA (i.e., aldosterone-producing adenoma (APA) or unilateral adrenal hyperplasia) and bilateral PA (i.e., bilateral adrenal hyperplasia). Adrenal vein sampling (AVS) is the most reliable procedure used to identify surgically curable PA [1]. Generally, use of adrenocorticotropic hormone (ACTH) stimulation during AVS is recommended to improve the AVS success rate [2]. However, numerous criteria for AVS, with and without ACTH stimulation, have been used to define unilateral versus bilateral PA [3], and several studies have reported diagnostic discrepancies in the final diagnosis obtained before versus after ACTH stimulation [4, 5].

AVS results are influenced by various factors such as the AVS protocol or technique. Several studies have revealed that APAs frequently harbor somatic mutations in *KCNJ5* [6], *ATP1A1* [7]*, ATP2B3* [7], and *CACNA1D* [8]. Seccia *et al.* reported that APAs with somatic *KCNJ5* mutations had greater aldosterone production on pre-ACTH AVS than did those without mutations [9]. Furthermore, new techniques using a monoclonal mouse anti-CYP11B2 antibody can elucidate the condition of the adjacent adrenal gland. Monticone *et al.* showed that the lateralization index (LI) was higher in the subgroup of APA patients without aldosterone-producing cell clusters (APCCs) in the adjacent adrenal cortex compared with the subgroup with APCCs using CYP11B2 immunostaining [10]. However, the relationship between genetic and immunohistochemical analysis results and the diagnostic discrepancy between pre- and post-ACTH has not been well elucidated.

Therefore, we investigated the influence of genetic and immunohistochemical characterization of PA on the diagnostic discrepancy between pre- and post-ACTH AVS. This was achieved using immunohistochemical and genetic analyses of surgical specimens.

## RESULTS

### Success rate of AVS pre- versus post-ACTH

The AVS success rate was 87% (169 of 195 patients) for pre-ACTH AVS, 91% (177 of 195 patients) for post-ACTH AVS, and 81% (158 of 195 patients) for both pre- and post-ACTH AVS (Table 1, Supplementary Table 1). The success rate of post-ACTH AVS was equivalent to that of pre-ACTH AVS.

**Table 1:** Success rate of adrenal vein sampling pre- and post-ACTH stimulation

	Pre-ACTH [*n* (%)]	Post-ACTH [*n* (%)]	Both pre- and post-ACTH [*n* (%)]
All (*n* = 195)	169 (87)	177 (91)	158 (81)
Pre-ACTH using QCA (*n* = 105)	96 (91)	93 (89)	86 (82)
Pre-ACTH without using QCA (*n* = 90)	73 (81)	84 (93)	72 (80)

QCA, quick cortisol assay.

### Diagnosis and treatment strategy according to pre- and post-ACTH AVS

**Figure 1:**
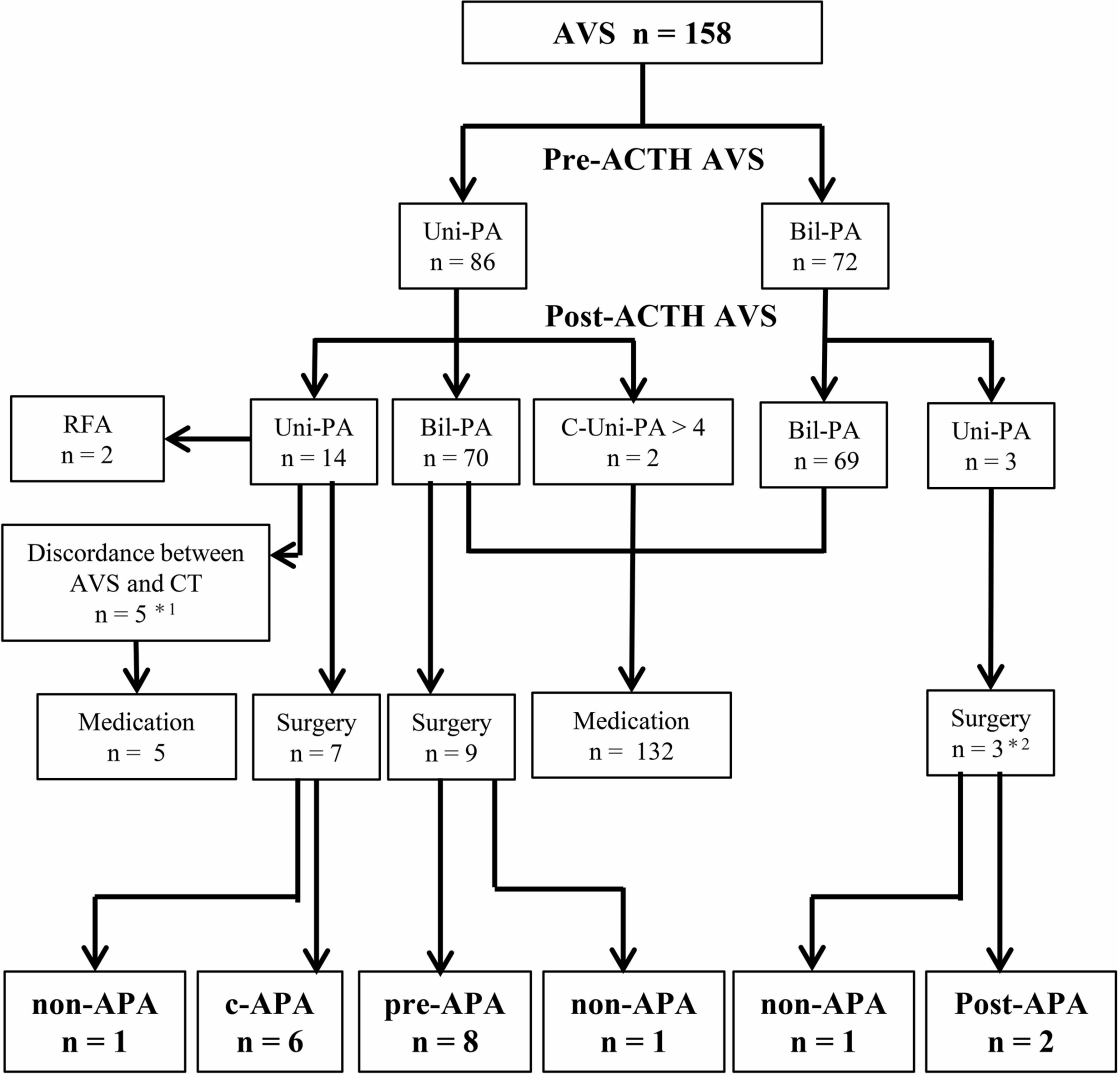
Treatment strategy decision according to adrenal vein sampling Lateralization was determined according to guidelines of the Endocrine Society. ^*^1 patients with discordant findings between computed tomography and AVS who were treated with an mineralocorticoid receptor antagonist and whose blood pressure was under control. ^*^2 patients whose diagnosis changed to unilateral PA after ACTH stimulation; nevertheless, they underwent adrenalectomy because of severe hypokalemia (*n* = 2) or resistant hypertension (*n* = 1). AVS, adrenal vein sampling. Uni-PA, unilateral PA. Bil-PA, bilateral PA. C-Uni-PA, patients whose diagnosis changed to contralateral unilateral PA after ACTH stimulation. RFA, radiofrequency ablation. c-APA, concordant aldosterone-producing adenoma (APA) group (i.e., patients diagnosed with APA by both pre- and post-ACTH). pre-APA, pre-APA group (i.e., patients diagnosed with APA by pre-ACTH only). post-APA, post-APA group (i.e., patients diagnosed with APA by post-ACTH only. non-APA, patients without APA.

We analyzed the frequency of a change in diagnosis from pre- to post-ACTH AVS, as well as the impact of AVS on the treatment strategy decision (Figure 1). The diagnoses determined by pre- and post-ACTH AVS were in accordance in 83 (53%) of the 158 patients with successful pre- and post-ACTH AVS: unilateral PA in 14 and bilateral PA in 69 (Table 2). Of the 14 unilateral PA cases, 9 underwent laparoscopic adrenalectomy or radiofrequency ablation, and 5 were prescribed medication because of a discordance between the lateralization of the adrenal tumor and AVS. A diagnostic discrepancy between pre- and post-ACTH AVS was observed in 75 of the 158 patients. Of 70 patients whose diagnosis changed to bilateral PA after ACTH stimulation, 9 underwent laparoscopic adrenalectomy. Two patients whose diagnosis changed to contralateral aldosterone overproduction after ACTH stimulation were prescribed medication. Three patients whose diagnosis changed to unilateral PA after ACTH stimulation underwent laparoscopic adrenalectomy.

**Table 2:** Comparative analysis of the final diagnosis pre- versus post-ACTH adrenal vein sampling

Lateralization (All, *n* = 158)		Post-ACTH LI > 4	
		Unilateral (*n*)	Bilateral (*n*)
Pre-ACTH	Unilateral	14	70
LI > 2	Bilateral	3	69

In 2 of the 158 cases (omitted from the table), the diagnosis was changed to contralateral aldosterone overproduction post-ACTH stimulation.

### Postoperative evaluation

We examined the effect of laparoscopic adrenalectomy on patients with PA, using the criteria of the Primary Aldosteronism Surgical Outcome study [11] (Supplementary Table 2). Of nine patients diagnosed with unilateral PA by both pre- and post-ACTH AVS, seven underwent unilateral adrenalectomy, of whom six patients obtained complete or partial clinical success and complete biochemical success (Supplementary Figure 1). Of nine patients diagnosed with unilateral PA by pre-ACTH AVS only and who underwent unilateral adrenalectomy, eight obtained complete or partial clinical success and complete biochemical success (Supplementary Figure 1).

### Detection of the lesion responsible for PA by CYP11B2 immunostaining

**Figure 2:**
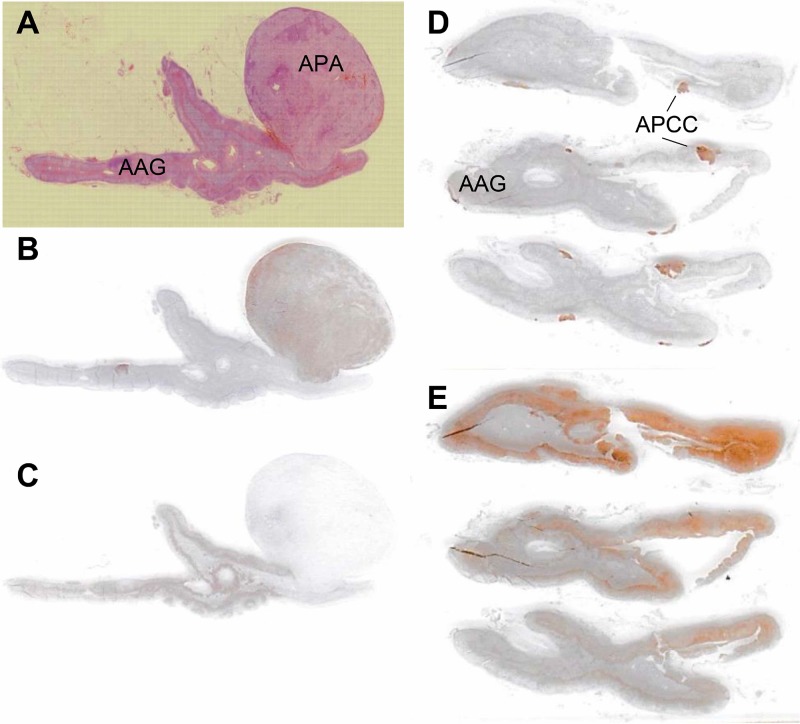
Histopathological and immunohistochemical analyses of CYP11B2 expression in aldosterone-producing adenomas and adjacent adrenal glands (**A**–**C**: case 11; **D**, **E**: case 9). Formalin-fixed paraffin-embedded tissue sections were stained with hematoxylin and eosin (A), anti-CYP11B2 antibody (B, D), and anti-CYP11B1 antibody (C, E). APA, aldosterone-producing adenoma; AAG, adjacent adrenal gland; APCC, aldosterone-producing cell cluster.

In this study, 19 PA patients underwent both pre- and post-ACTH AVS successfully, followed by unilateral adrenalectomy. We attempted to identify the lesion responsible for aldosterone overproduction by immunostaining for CYP11B2. The adjacent adrenal gland was examined for the presence of APCCs, defined as cell clusters expressing CYP11B2 but not CYP11B1 immunohistochemically [12] (Figure 2). Of the 19 patients, 16 had CYP11B2-positive adenomas, which were considered APAs (Supplementary Table 3). One patient with a CYP11B2-negative tumor had multiple APCCs. Another two patients had multiple APCCs without tumors.

### Gene mutation analysis

We analyzed the 16 CYP11B2-positive APAs for gene mutations. In a genetic analysis, 11 of these 16 APA patients had a somatic mutation in the *KCNJ5* gene, and 5 had no known mutations in any gene.

### Histopathological analysis

According to hematoxylin and eosin staining, 13 of the 16 CYP11B2-positive APAs consisted of mainly zona fasciculata (ZF)-like cells, and the remaining 3 consisted of mainly zona glomerulosa (ZG)-like cells.

### Semi-quantitative analysis of CYP11B2 immunostaining

**Figure 3:**
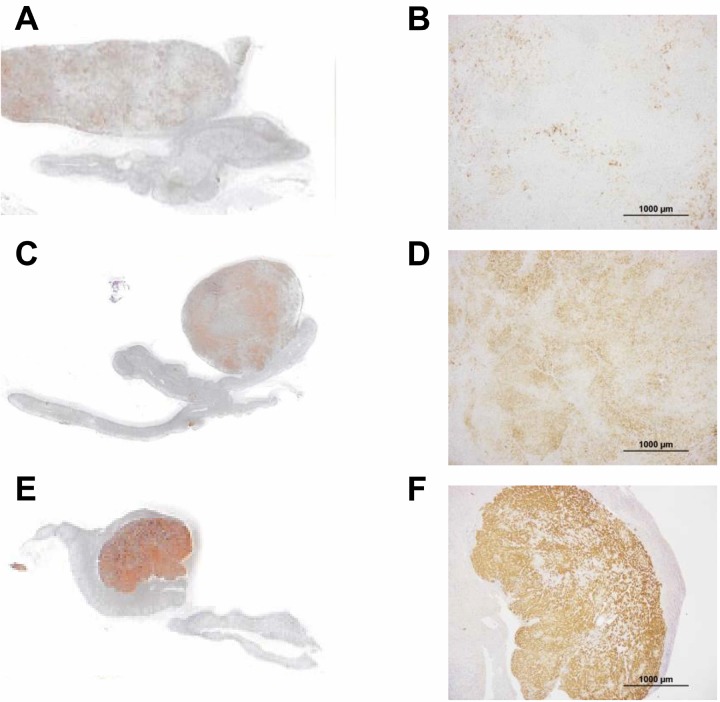
Semi-quantitative analysis of aldosterone-producing adenomas in tissues immunostained for CYP11B2 (**A**, **B**) case 5; (**C**, **D**) case 9; (**E**, **F**) case 2. Scale bars, 100 μm (B, D, and F). CYP11B2 staining was semi-quantitatively scored as 1, 2, and 3 in A, B, and C, respectively.

The intensity of CYP11B2 immunostaining in tumors was evaluated semi-quantitatively using a previously reported scoring system as follows: 1 = weak; 2 = intermediate; 3 = strong (Figure 3) [13]. In the semi-quantitative analysis of CYP11B2 staining, of the 16 CYP11B2-positive APAs, 8 had a score of 3, 4 a score of 2, and 4 a score of 1.

### Prevalence of APCCs in the adjacent adrenal gland

We examined the prevalence of APCCs in the adrenal gland adjacent to the APA. Among patients with APA, the prevalence of APCCs was 94% (15/16), and the median number of APCCs was 3.5 (interquartile range [IQR] 1.0–6.3).

### Relationship between the diagnostic discrepancy and the findings of immunohistochemical and genetic analyses

Among the 16 patients with CYP11B2-positive APAs, 10 showed a discrepancy between their pre- and post-ACTH AVS diagnoses. The 16 patients were divided into three subgroups (Table 3): the concordant APA (c-APA) group (*n* = 6), which included patients diagnosed with APA by both pre- and post-ACTH, the pre-APA group (*n* = 8), which included patients diagnosed with APA by pre-ACTH only, and the post-APA group (*n* = 2), which included patients diagnosed with APA by post-ACTH only. PA patients without APA (*n* = 3) were classified into the non-APA group.

**Table 3:** Adrenal vein sampling and histopathological findings of patients with aldosterone-producing adenoma

Group	Case	Age/Sex	PAC (pg/dL)	PRA (ng/mL/h)	Pre-ACTH		Post-ACTH		CT findings	Tumor size (mm)	Cell composition	CYP11B2 staining	Gene mutation	ATII responsiveness	Clinical success	Biochemical success
					LI	CR	LI	CR								
c-APA	1	62/M	82	0.4	2.9	0.8	9.8	0.6	Negative	5	ZF	2	KCNJ5 p.T158A	Responsive	Complete	Complete
	2	40/M	149	0.3	26.7	1.2	4.1	0.4	Tumor	12	ZG	3	WT	ND	Partial	Complete
	3	50/M	475	0.9	23.0	0.4	21.9	0.3	Tumor	14	ZF	3	KCNJ5 p.G151R	ND	Partial	Complete
	4	59/M	255	0.7	15.9	0.2	9.7	0.3	Tumor	13	ZF	2	KCNJ5 p.G151R	ND	Partial	Complete
	5	53/F	970	0.3	12.3	0.1	16.3	0.1	Tumor	23	ZF	1	KCNJ5 p.L168R	ND	Complete	Complete
	6	42/F	413	0.2	25.2	1.2	14.3	0.4	Tumor	18	ZF	3	*KCNJ5* p.G151R	ND	Complete	Complete
pre-APA	7	50/F	107	0.2	2.6	0.5	2.3	1.7	Tumor	12	ZF	1	KCNJ5 p.L168R	ND	Partial	Complete
	8	69/F	114	0.3	10.9	0.3	3.2	0.7	Tumor	14	ZF	3	KCNJ5 p.L168R	Responsive	Partial	Complete
	9	50/M	127	0.2	2.4	2.5	2.5	0.9	Negative	3	ZF	3	WT	ND	Absent	Absent
	10	54/M	147	0.5	38.9	0.6	3.9	1.2	Tumor	8	ZF	1	KCNJ5 p.G151R	ND	Partial	Complete
	11	33/M	360	0.6	2.7	0.3	2.5	0.3	Tumor	15	ZF	2	KCNJ5 p.G151R	Responsive	Partial	Complete
	12	33/M	284	0.3	8.3	0.1	3.6	0.2	Tumor	16	ZF	2	KCNJ5 p.L168R	Responsive	Complete	Complete
	13	67/F	231	0.7	4.0	0.4	2.6	0.9	Negative	5	ZG	3	WT	ND	Partial	Complete
	14	37/M	176	0.2	4.4	0.6	3.1	1.1	Tumor	6	ZF	3	*KCNJ5* p.G151R	ND	Complete	Complete
post-APA	15	65/M	166	0.3	0.7	0.4	8.6	0.5	Negative	5	ZG	3	WT	Responsive	Partial	Complete
	16	62/M	290	0.5	1.2	0.5	5.8	1.6	Negative	7	ZF	2	WT	ND	Partial	Partial
non-APA	17	57/F	142	0.4	2.5	1.0	11.5	0.3	Negative	-	-	-	-	ND	Absent	Absent
	18	52/F	175	0.5	5.1	0.5	3.0	1.4	Tumor	-	-	-	-	ND	Partial	Complete
	19	81/F	81	0.2	1.8	0.9	4.4	1.6	Tumor	-	-	-	-	ND	Partial	Complete

PAC, plasma aldosterone concentration; PRA, plasma renin activity; ACTH, adrenocorticotropic hormone; LI, lateralization index; CR, contralateral ratio; APA, aldosterone-producing adenoma; M, male; F, female; ZF, zona fasciculata-like cell type; ZG, zona glomerulosa-like cell type; WT, wild type; ATII, angiotensin II; ND, no data.

In the genetic analysis, 5 of 6 patients in the c-APA group and 6 of 8 in the pre-APA group had a somatic mutation in *KCNJ5.* Neither patient in the post-APA group had known mutations in any gene. The frequency of the patients with *KCNJ5* mutations was lower in the pre-APA group than in the c-APA group, but not significantly so (75% vs. 83%).

In a pathological analysis, tumor size was not significantly different among the three APA subgroups. In terms of cell composition, 5 of the 6 APAs in the c-APA group and 7 of the 8 in the pre-APA group were composed mainly of ZG-like cells. The proportion of APAs composed mainly of ZG-like cells was higher in the pre-APA group than in the c-APA group, but not significantly so (88% vs. 83%).

**Figure 4:**
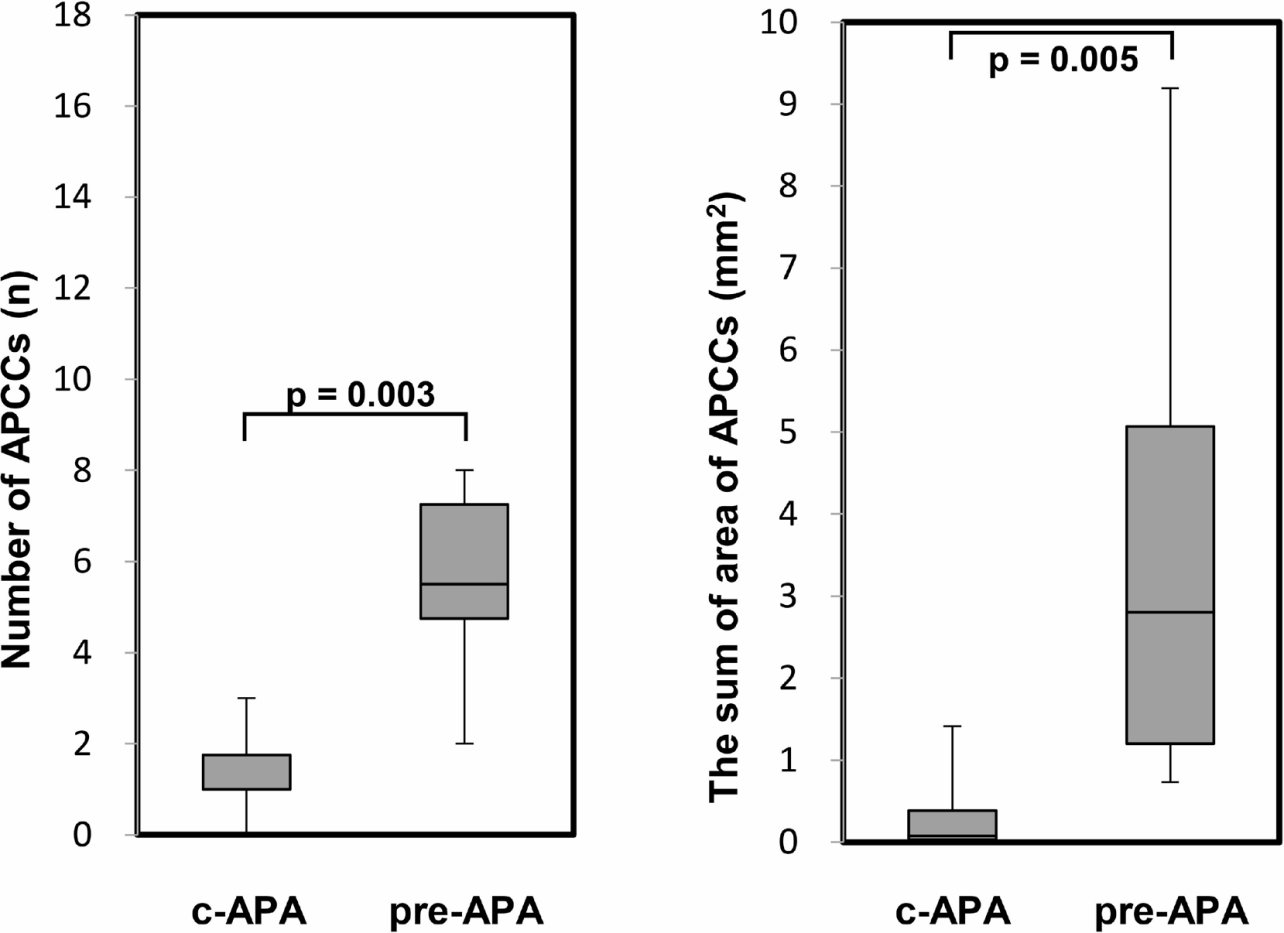
The number and the summed area of APCC(s) in adrenal tissue adjacent to the APA PA patients were divided into the following four subgroups (Table 3): c-APA group (*n* = 6), which included patients diagnosed with APA by both pre- and post-ACTH; pre-APA group (*n* = 8), which included patients diagnosed with APA by pre-ACTH only. Data are shown as box plots and were analyzed using the Mann–Whitney *U* test.

**Figure 5:**
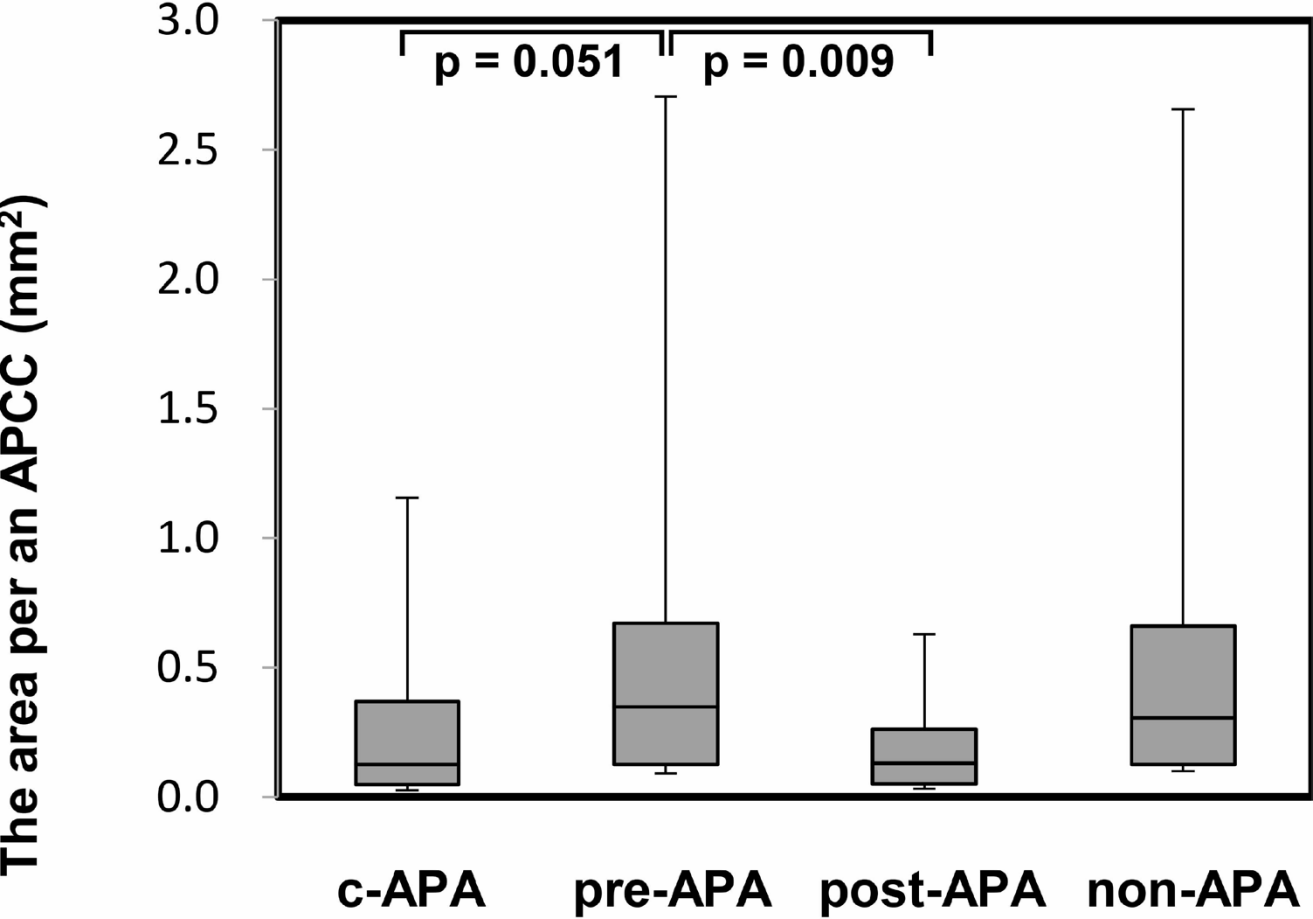
The area per an APCC in adrenal tissue adjacent to the APA PA patients were divided into the following four subgroups (Table 3): c-APA group (*n* = 6, 8 total APCCs); pre-APA group (*n* = 8, 45 total APCCs); post-APA group (*n* = 2, 10 total APCCs), which included patients diagnosed with APA by post-ACTH only; and the non-APA group (*n* = 3, 31 total APCCs), which included PA patients without APA. Data are shown as box plots and were analyzed using the Mann–Whitney *U* test.

In the immunohistochemical analysis, the CYP11B2 staining level did not show a significant difference among the three subgroups. However, the number of APCCs in adrenal tissue adjacent to the APA was significantly greater in the pre-APA group (median 5.5, IQR 4.8–7.3) than in the c-APA group (median 1.0, IQR 1.0–1.8) (*p* = 0.003) (Figure 4). In addition, the summed APCC area was also significantly greater in the pre-APA group (median 1.60, IQR 1.20–2.27 mm^2^) than in the c-APA group (median 0.05, IQR 0.03–0.31 mm^2^) (*p* = 0.005) (Figure 4). Furthermore, the area per APCC was significantly greater in the pre-APA group (median 0.22, IQR 0.13–0.32 mm^2^) than in the post-APA group (median 0.08, IQR 0.05–0.13 mm^2^) (*p* = 0.009) (Figure 5).

### Angiotensin II responsiveness of APA

Among the 16 patients with CYP11B2-positive APAs, 5 patients were examined with furosemide and upright posture test. All five patients were categorized as Angiotensin II (ATII)-responsive APA because of an increase of PAC after the furosemide and upright posture test.

### The ratio of post- to pre-ACTH plasma aldosterone or cortisol concentrations

**Figure 6:**
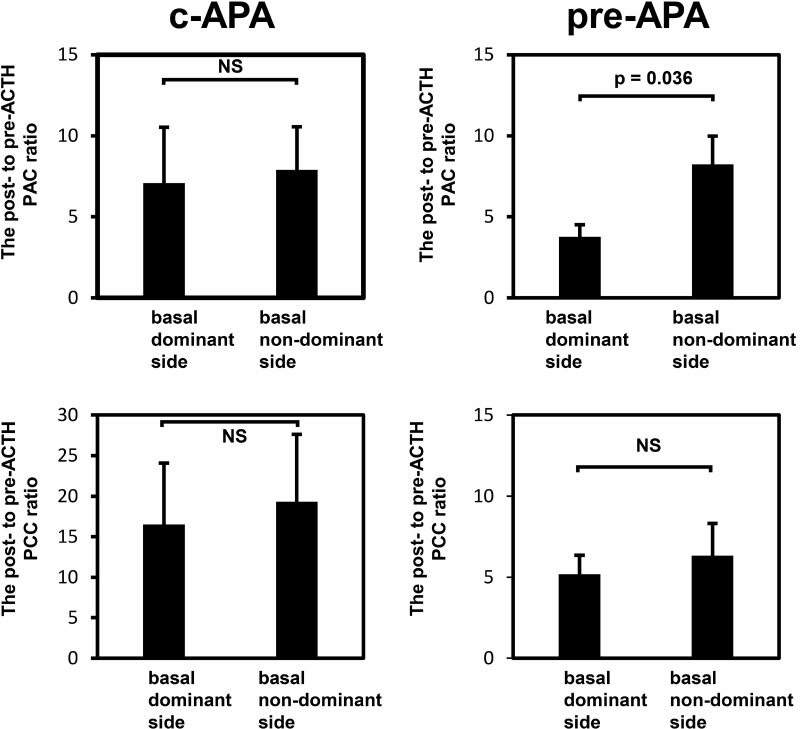
The post- to pre-ACTH plasma aldosterone or cortisol concentration ratio Data are shown as means ± SEM and were analyzed using the Wilcoxon signed-rank test.

In the c-APA group, the ratios of post- to pre-ACTH plasma aldosterone concentrations (PACs) (7.1 vs. 7.9) and plasma cortisol concentrations (16.5 vs. 19.3) did not differ between the basal dominant and nondominant sides (Figure 6). In the pre-APA group, the post- to pre-ACTH plasma cortisol concentration ratio did not differ between the basal dominant and nondominant sides (5.2 vs. 6.3); however, the post- to pre-ACTH PAC ratio was significantly higher on the basal nondominant than dominant side (8.2 vs. 3.8, *p* = 0.036).

## DISCUSSION

In the present study, we demonstrated a discrepancy in the final diagnoses obtained by pre- versus post-ACTH AVS in PA patients as reported previously. However, unilateral adrenalectomy, performed based on pre-ACTH AVS criteria, resulted in a high cure rate for PA. Furthermore, immunohistochemical analysis revealed that both the number and summed area of APCCs were significantly smaller in the c-APA group, which comprised patients diagnosed with APA by both pre- and post-ACTH AVS, than in the pre-APA group, which comprised patients diagnosed with APA by pre-ACTH AVS only.

The influence of ACTH stimulation on PA laterality is controversial. Several studies have reported that ACTH stimulation causes some discrepancy in the final diagnosis between pre- and post-ACTH AVS [4, 5]. First, we considered the different ACTH stimulation methods used. Seccia *et al.* used three different AVS protocols and reported some discrepancies between bolus and continuous ACTH stimulation methods [14]. Second, we considered the roles of the AVS criteria applied. Monticone *et al.* indicated that a higher concordance between the pre- and post-ACTH AVS diagnoses was achieved when strict cannulation criteria were applied [15]. However, their results were different from ours (Supplementary Table 4). Even when we applied strict selectivity index criteria, the number of patients excluded from this study was still small. These results suggest that the diagnostic discrepancy caused by ACTH stimulation might be unrelated to the ACTH stimulation protocol or AVS technique.

Most APAs are considered to be responsive to ACTH stimulation [16]. Our results showed that ACTH stimulation decreased the LI in 81% of patients (127/158), and the diagnosis of approximately 40% of patients changed to bilateral PA after ACTH stimulation. These results suggest that PA patients may have APAs unresponsive to ACTH. Another factor that stimulates aldosterone synthesis is angiotensin II (ATII), and some APAs display a response to ATII [17]. The PA patients whose diagnosis changed to bilateral PA after ACTH stimulation may have had APAs responsive to ATII, and ACTH stimulation might have induced aldosterone production in the contralateral adrenal gland, leading to a reduction in the LI. However, a previous study showed that ATII-responsive APAs also responded to ACTH infusion [18]. In our study, based on our criteria for ATII-responsive APA [17], all five APAs examined by the furosemide plus upright test were responsive to ATII. Thus, according to this result, ATII responsiveness may be unrelated to the diagnostic discrepancy. Recently, El Ghorayeb *et al.* reported that melanocortin-2 receptor (MC2R), also known as ACTH receptor, may be responsible for aldosterone hypersecretion in APAs, and that the APA expression level of MC2R may be related to discordant results in AVS [19]. Our study showed that increased aldosterone secretion in the contralateral gland after ACTH stimulation decreased the LI of the patients whose diagnosis changed to bilateral PA. Our histopathological data suggested that aldosterone secretion from the adjacent gland post-ACTH stimulation may be related to the existence of APCCs. In the post-APA group (*n* = 2), the post- to pre-ACTH PAC ratio was higher on the dominant (mean 30.4) than nondominant (mean 4.9) side post-ACTH. PA patients in the post-APA group may have possessed aldosteronomas with high MC2R expression. A change in diagnosis post-ACTH stimulation to bilateral PA may be associated with the presence of multiple APCCs, while that to unilateral PA may be associated with high MC2R expression. Unfortunately, the number of cases with available flash-frozen specimens was very small in this study. We are planning a further study on the expression of MC2R in APA and the adrenal gland.

Previous studies showed that ACTH responsiveness is associated with somatic mutations in APA [9]. In an *in vitro* study, Kitamoto *et al.* reported that the responsiveness of aldosterone production to ACTH is higher in both *ATP2B3*- and *CACNA1D*-mutated APAs than in wild-type APAs [20]. In this study, no patients had APA with these mutations, while most had *KCNJ5* mutations, although the number of surgical cases was small. A recent meta-analysis in Western countries showed that young age, female sex, and larger tumors are the phenotypic features of APA patients with *KCNJ5* mutations [21]. Although the number of PA patients was small in this study, the size of tumors harboring mutated *KCNJ5* (*n* = 11) was larger than that of wild-type *KCNJ5* tumors (*n* = 5) (13.1 vs. 6.4, *p* = 0.009). However, we did not find any significant differences in age, sex, or laboratory data in patients with mutant versus wild-type *KCNJ5* tumors. Furthermore, a previous study reported that *KCNJ5* mutations are associated with a higher LI, thereby influencing AVS results [9]. In this study, the LI was higher in APA patients with somatic *KCNJ5* mutations than in those without mutations on both pre- (13.4 vs. 7.0, *p* = 0.06) and post-ACTH AVS (8.2 vs. 4.7, *p* = 0.17), but not significantly. In addition, there was no significant difference in the proportion of patients with *KCNJ5* mutations between the c-APA and pre-APA groups. These results suggest that somatic mutations in APA might not be related to the diagnostic discrepancy. Previous reports showed that the CYP11B2 H-score in APAs displayed a positive correlation with plasma aldosterone levels and the ARR, which reflect the biochemical severity of PA [13]. We investigated the relationship between the CYP11B2 immunostaining level in APAs and the AVS findings. However, there was no significant correlation.

CYP11B2 immunostaining can also be used to reveal adrenocortical pathological conditions including the presence of APCCs, which are cell clusters exhibiting a variegated pattern and high expression of CYP11B2 [12]. A recent study showed that aging and certain environmental agents are associated with APCC development, and that APCCs are frequently seen in nonhypertensive human adrenal glands [22]. As a consequence, APA patients with multiple APCCs are presumed to have the same adrenocortical conditions in the opposite adrenal gland. This assumption was supported by our finding that the post- to pre-ACTH PAC ratio was significantly higher on the non-dominant than dominant side in the pre-APA group harboring multiple APCCs (Figure 6). Even though the aldosterone responsiveness to ACTH of APCCs has not been fully elucidated, it is conceivable that ACTH stimulation induces aldosterone production by APCCs in the opposite adrenal gland, leading to a decreased LI. Recently, segmental AVS, in which samples of adrenal effluent are collected from tributaries of draining adrenal segments using coaxial microcatheters, has been performed in some institutions [23]. This method can be used to detect and localize intra-adrenal aldosterone secretion. Segmental AVS may be useful in PA patients with discordant results to demonstrate aldosterone secretion from APCCs.

At our institution, the treatment policy for PA patients with discordant results on AVS is pharmacotherapy, using mineralocorticoid receptor (MR) antagonists as the first choice, rather than surgery. Adrenalectomy is recommended over pharmacotherapy only for PA patients with resistant hypertension, severe hypokalemia, or progression of atherosclerosis and cardiovascular disease despite pharmacotherapy. This is because no compelling evidence indicates that adrenalectomy is superior to MR antagonists in terms of improving prognosis and long-term organ damage. In addition, although rare, APA cured by long-term administration of an MR antagonist has been reported [24]. Furthermore, patients with discordant findings between computed tomography and AVS were also treated with MR antagonists in this study (Figure 1). Tumors with no aldosterone overproduction are at risk of being malignant. It is necessary to be determined in consideration of the individual situation.

According to our criteria, 16 of 19 surgical cases achieved a biochemical cure even when we included discordant cases: 6/6 in c-APA, 7/8 in pre-APA, 1/2 in post-APA, and 2/3 in non-APA groups. When we used PA biochemical cure as the reference gold standard, the number of patients that surgery was not effective was three: one in the pre-APA, one in the post-APA, and one in the non-APA group. These patients had in common a low LI on pre-ACTH AVS (range 1.2–2.5), an increased LI on post-ACTH AVS, and no or undetectable tumors on adrenal CT. Although this analysis included only a small minority of the patients, our study suggested that PA patients with bilateral normal CT findings and an LI < 2.5 on pre-ACTH AVS should be treated medically, even if the LI is higher on post- than pre-ACTH AVS or is > 4.

In conclusion, a diagnostic discrepancy between pre- and post-ACTH AVS is possible and potentially caused by the presence of APCCs. The existence of APCCs, which may affect the lateralization diagnosis on AVS, and the efficacy of adrenalectomy based on pre-ACTH AVS criteria suggest that pre-ACTH AVS is more important for the treatment strategy decision.

## MATERIALS AND METHODS

### AVS criteria pre- and post-ACTH stimulation

The criterion for successful selective catheterization was a ratio of adrenal vein to inferior vena cava cortisol concentrations (i.e., selectivity index > 2 for pre-ACTH AVS and > 5 for post-ACTH AVS [25]). The LI is defined as the ipsilateral adrenal vein aldosterone to cortisol concentration ratio over the contralateral aldosterone to cortisol ratio. In pre-ACTH AVS, unilateral aldosterone overproduction was confirmed by a LI > 2. In post-ACTH AVS, unilateral aldosterone overproduction was confirmed by a LI > 4 [25]. We examined the success rate and diagnostic discrepancy of AVS with versus without ACTH stimulation in both patient subsets.

### Patients

In this study, a total of 195 patients were diagnosed with PA and underwent both pre- and post-ACTH AVS at Kanazawa University Hospital from June 2011 to September 2017 (Supplementary Table 1). Of these, 158 patients underwent successful pre- and post-ACTH AVS, of whom 75 patients had a discrepancy in their final diagnosis between pre- and post-ACTH. As a consequence, 19 of these patients underwent unilateral adrenalectomy.

All patients were evaluated after discontinuing all antihypertensive drugs except for calcium channel blockers and alpha blockers, for 2 weeks, or 6 weeks if using spironolactone and eplerenone, before screening. Patients taking several antihypertensive drugs or who had high BP and were not taking medication were allowed to take an α-blocker and/or a calcium channel blocker and were maintained on the same drug until finishing AVS. PA was diagnosed based on a PAC (pg/mL) to plasma renin activity (ng/mL/h) ratio of >200. The diagnosis was confirmed by a captopril challenge test, furosemide plus upright test, and/or saline infusion test [26]. All patients with an adrenal tumor underwent a dexamethasone suppression test for the exclusion of Cushing's syndrome. Clinical and biochemical outcomes were evaluated using the criteria of the Primary Aldosteronism Surgical Outcome study [11] (Supplementary Table 2).

### AVS technique

All patients in this study underwent AVS at Kanazawa University Hospital. All were subjected to computed tomography before undergoing AVS. AVS was started in the morning and performed using simultaneous bilateral catheterization pre- and post-ACTH stimulation. Following pre-ACTH AVS, post-ACTH AVS was performed by bolus injection of 250 μg synthetic ACTH. From 2011 to 2014, pre-ACTH AVS was performed using a semiquantitative quick cortisol assay as described previously [27]. The cortisol concentration obtained by the semiquantitative quick cortisol assay was used for assessment of catheterization during AVS only, while we used the cortisol concentration measured by the CLIA method (BML, Tokyo, Japan) in our clinical laboratory department for the final assessment of catheterization and lateralization.

### Histopathological and immunohistochemical analysis

All pathological specimens were prepared according to the General Rule for Clinical and Pathological Studies on Adrenal Tumor in Japan. All adrenal glands included in the study were paraffin embedded and stained with hematoxylin and eosin. Immunohistochemical staining was performed using anti-CYP11B1 and -CYP11B2 antibodies (kind gifts from Dr. Celso E. Gomez-Sanchez in Endocrinology Section, G.V. (Sonny) Montgomery VA Medical Center and University of Mississippi Medical Center, Jackson, MS 39216, USA.) using ChemMate ENVISION kits (DAKO, Glostrup, Denmark). The specificities of the anti-CYP11B1 and -CYP11B2 antibodies were established in a previous study [28]. We defined the CYP11B2-positive area as the lesion responsible for aldosterone overproduction (Figure 3). Cellular composition was determined by examining for known features of the ZF (large lipid-laden clear cells with round to oval vesicular nuclei) and ZG (small compact cells with a high nuclear/cytoplasmic ratio and moderate lipid levels) by hematoxylin and eosin staining [10, 29, 30]. We measured ZF-like and ZG-like cell and APCCs' areas using ImageJ. We counted the number of APCCs in all formalin-fixed paraffin-embedded tissue sections.

### DNA extraction and gene mutation analysis

We analyzed the presence of specific gene mutations in the lesion responsible for aldosterone overproduction. DNA was extracted from formalin-fixed paraffin-embedded tissue sections using the Gentra Puregene Tissue Kit (QIAGEN, Hilden, Germany). Mutation analysis of *KCNJ5*, *ATP1A1*, *ATP2B3*, and *CACNA1D* gene was performed using specific primers (Supplementary Table 5) [31]. Direct sequencing was performed using the ABI PRISM 310 Genetic Analyzer (Thermo Fisher Scientific, Bremen, Germany).

### Aldosterone responsiveness of APA to ATII

Previously reported criteria for measuring the aldosterone responsiveness of APA to ATII were used [17]. ATII responsiveness in APA was defined as an increase in the PAC relative to the baseline value after administration of 40 mg furosemide followed by standing for 2 h.

### Plasma aldosterone and cortisol concentrations in the adrenal vein post-ACTH stimulation

The ratios of the post- to pre-ACTH PAC or plasma cortisol concentration in the adrenal vein were calculated. We compared the ratios between the basal dominant and non-dominant sides. The basal dominant side indicates the side on which pre-ACTH AVS showed aldosterone overproduction.

### Statistics and ethics

Data are expressed as means ± standard error of the mean. Statistical analysis was performed using Excel 2016 (Microsoft, Seattle, WA, USA) with the add-in software Statcel4 (OMS, Tokyo, Japan). The Mann–Whitney *U* test was used to compare differences between two independent groups and the Wilcoxon signed-rank test to compare differences between two related samples. Values of *P* < 0.05 were considered statistically significant.

This clinical study was approved by the ethics committees of Kanazawa University (no. 2015121). The genetic experiment was approved by the ethics committees of Kanazawa University (no. 2012013 and 2012019) and Houju Memorial Hospital (no. 18-3 and 18-4). The immunohistochemical analysis was approved by the ethics committees of Saitama Medical University International Medical Center (no. 16-093).

## SUPPLEMENTARY MATERIALS FIGURES AND TABLES


