# Supplementary material for: Impact of aldosterone-producing cell clusters on diagnostic discrepancies in primary aldosteronism
Source: Oncotarget. 2018 May 25;9(40):26007–18. doi: 10.18632/oncotarget.25418 (PMC5995250; doi:10.18632/oncotarget.25418)
Supplement: Supplementary file 1 [file oncotarget-09-26007-s001.pdf]

Impact of aldosterone-producing cell clusters on diagnostic discrepancies in primary aldosteronism

SUPPLEMENTARY MATERIALS

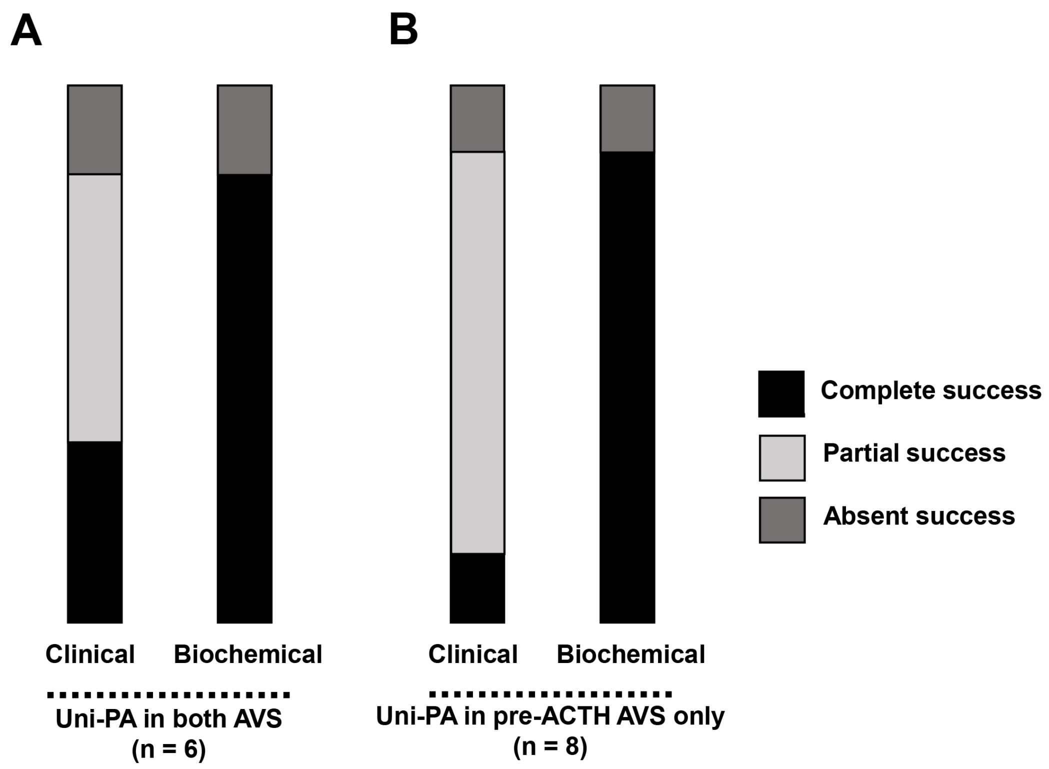

Supplementary Figure 1: Clinical and biochemical outcomes after unilateral adrenalectomy in Uni-PA in both AVS (**A**) and Uni-PA in only pre-ACTH AVS (**B**) Clinical (left) and biochemical (right) outcomes were assessed in accordance with the criteria of the Primary Aldosteronism Surgical Outcome (PASO) study (Supplementary Table 1). Uni-PA, unilateral PA.

Supplementary Table 1: Summary of clinical characteristics of patients with primary aldosteronism

	All PA patients	PA patients with successful both AVS
Number of cases, *n*	195	158
Age, y [mean (range)]	54 (24–81)	54 (24–81)
Sex, males/females	101/94	82/76
Systolic blood pressure, mmHg	137 ± 17	137 ± 17
Diastolic blood pressure, mmHg	85 ± 12	86 ± 12
Plasma aldosterone concentration, pg/mL	182 ± 193	166 ± 103
Plasma renin activity, ng/mL/h	0.4 ± 0.3	0.4 ± 0.2
Aldosterone renin ratio	712 ± 1129	599 ± 638
Serum potassium concentration, mEq/L	3.9 ± 0.4	3.9 ± 0.4

Data presented as mean ± SD, except where noted otherwise.

Supplementary Table 2: International consensus on surgery outcomes for unilateral primary aldosteronism by the Primary Aldosteronism Surgical Outcome (PASO) study

	Clinical	Biochemical
Complete success	Normal blood pressure without the aid of antihypertensive medication	Correction of hypokalaemia (if present pre- surgery) and normalisation of the aldosterone-to- renin ratio; in patients with a raised aldosterone- to-renin ratio post-surgery, aldosterone secretion should be suppressed in a confirmatory
Partial success	The same blood pressure as before surgery with less antihypertensive medication or a reduction in blood pressure with either the same amount or less antihypertensive medication	test Correction of hypokalaemia (if present pre- surgery) and a raised aldosterone-to-renin ratio with one or both of the following (compared with pre-surgery): ≥50% decrease in baseline plasma aldosterone concentration; or abnormal but improved post-surgery
Absent success	Unchanged or increased blood pressure† with either the same amount or an increase in antihypertensive medication	confirmatory test result Persistent hypokalaemia (if present pre-surgery) or persistent raised aldosterone-to-renin ratio, or both, with failure to suppress aldosterone secretion with a post-surgery confirmatory test

(Williams TA *et al*. Outcomes after adrenalectomy for unilateral primary aldosteronism: an international consensus on outcome measures and analysis of remission rates in an international cohort.

Lancet Diabetes Endocrinol. 2017.

Supplementary Table 3: CYP11B2 staining of APA and pre-surgical treatment

Group	Case	CYP11B2 staining	Pre-surgical treatment
c-APA	1	CYP11B2-positive tumor	Amlodipine
	2	CYP11B2-positive tumor	Eplerenone, Amlodipine
	3	CYP11B2-positive tumor	Eplerenone, Amlodipine
	4	CYP11B2-positive tumor	Eplerenone, Amlodipine
	5	CYP11B2-positive tumor	Eplerenone
	6	CYP11B2-positive tumor	Eplerenone, Amlodipine
pre-APA	7	CYP11B2-positive tumor	Eplerenone, Adalat, Doxazosin
	8	CYP11B2-positive tumor	Cilnidipine, Doxazosin
	9	CYP11B2-positive tumor	Eplerenone, Amlodipine
	10	CYP11B2-positive tumor	Eplerenone, Amlodipine
	11	CYP11B2-positive tumor	Eplerenone, Amlodipine
	12	CYP11B2-positive tumor	Amlodipine
	13	CYP11B2-positive tumor	Amlodipine
	14	CYP11B2-positive tumor	Eplerenone, Amlodipine
post-APA	15	CYP11B2-positive tumor	Cilnidipine, Adalat, Doxazosin
	16	CYP11B2-positive tumor	Adalat, Doxazosin
non-APA	17	No tumor	None
	18	CYP11B2-negative tumor	Eplerenone, Cilnidipine
	19	No tumor	Spironolactone

Supplementary Table 4: Change of the lateralization in each criteria

	LI >2 in pre-ACTH	LI >3 in pre-ACTH	LI >4 in pre-ACTH
Uni in pre-ACTH (*n*)	89	43	30
Diagnostic change in post-ACTH (*n*)	70 Uni → Bil	30 Uni → Bil	18 Uni → Bil
	3 Bil → Uni	3 Bil → Uni	3 Bil → Uni

Uni, unilateral PA; Bil, bilateral PA.

Supplementary Table 5: The primers used for DNA mutation analysis

Gene	Forward primer	Reverse primer
KCNJ5	CGACCAAGAGTGGATTCCTT	AGGGTCTCCGCTCTCTTCTT
ATP1A1 exon (ex) 4	TATATTGCCTTGTAAGTGCTGG	GAAGTGGGAGACAAAGACGG
ATP1A1 ex 8	CGTGGCTTCCTTCAGGTTAG	AGAGTGTAACATTCGTGCAAGC
ATP2B3 ex 8	TTCTTCCCTCTTCCTGTCCC	TTCTTACCCCAGTTTCCGAG
CACNA1D ex 8A	TGACCCTCTCTCCTTATTAAATCC	AAAGCTTGTGTGGTCTTGGC
CACNA1D ex 8B	AGCTGCAACTGGGGCTC	GCAGCTAGGAGACACGCAG
CACNA1D ex 17	ATTGTTGGCCGCACGTAG	CAACTGTTGCAGGGCTCC
